# High-density lipoprotein cholesterol efflux capacity and incidence of coronary artery disease and cardiovascular mortality: a systematic review and meta-analysis

**DOI:** 10.1186/s12944-022-01657-3

**Published:** 2022-05-28

**Authors:** Wenke Cheng, Maciej Rosolowski, Julia Boettner, Steffen Desch, Alexander Jobs, Holger Thiele, Petra Buettner

**Affiliations:** 1 Department of Internal Medicine/Cardiology, Heart Center Leipzig at University of Leipzig, Struempellstr. 39, 04289 Leipzig, Germany; 2 Medical Faculty, University of Leipzig, Leipzig, Germany; 3 Institute of Medical Informatics, Statistics and Epidemiology, University Leipzig, Leipzig, Germany

**Keywords:** Cardiovascular mortality, Coronary artery disease, Cholesterol efflux capacity, High-density lipoprotein, Meta-analysis

## Abstract

**Background:**

The preventive effect of cholesterol efflux capacity (CEC) on the progression of atherosclerotic lesions has been confirmed in animal models, but findings in the population are inconsistent. Therefore, this meta-analysis aimed to systematically investigate the relationship of CEC with coronary artery disease (CAD) and cardiovascular mortality in a general population.

**Methods:**

Four electronic databases (PubMed, Embase database, Cochrane Library, Web of Science) were searched from inception to February 1st, 2022 for relevant studies, without any language restriction. For continuous variables, the mean and standard deviation (SD), maximum adjusted odds ratios (ORs), relative risks (RRs), or hazard ratios (HRs) and 95% confidence intervals (CIs) were extracted. The random-effects model was adopted to calculate the pooled results, and dose-response analyses were conducted. All pooled results were expressed by standardized mean difference (SMD) and ORs.

**Results:**

Finally, 18 observational studies were included. Compared with the non-CAD group, the CAD group (SMD -0.48, 95% CI − 0.66 to − 0.30; I^2^ 88.9%) had significantly lower CEC. In the high-CEC population, the risks of CAD (OR 0.52, 95% CI 0.37 to 0.71; I^2^ 81%) significantly decreased, and a linear negative dose-response was detected. However, an association between CEC and the risk of cardiovascular mortality was not found (OR 0.44, 95% CI 0.18 to 1.06; I^2^ 83.2%).

**Conclusions:**

This meta-analysis suggests that decreased CEC is strongly associated with the risk of CAD, independent of HDL-C level. However, a decreased CEC seems not to be related to cardiovascular mortality. Meanwhile, CEC is linearly negatively correlated with the risk of CAD.

**Supplementary Information:**

The online version contains supplementary material available at 10.1186/s12944-022-01657-3.

## Introduction

Coronary artery disease (CAD), a manifestation of atherosclerosis, results in stable angina, unstable angina, myocardial infarction (MI), or sudden cardiac death [1]. Currently, CAD remains the leading cause of morbidity and mortality worldwide [2]. Atherosclerotic plaques are the hallmark of CAD, and its formation is closely associated with dyslipidemia and inflammatory processes [2–4]. During early plaque formation, endothelial cell damage is followed by inflammatory infiltration and abnormal accumulation of cholesterol in macrophages can result in foam cell formation and eventually atherosclerotic plaques [5, 6]. Considering these pathomechanisms, high-density lipoprotein (HDL) has been extensively studied due to its abilities of simultaneous reverse cholesterol transport (RCT) and anti-inflammatory effects [7, 8].

RCT is the process where excess cholesterol is transported via HDL from peripheral tissues to the liver and ultimately to the bile and feces for excretion [9]. In 1975, it was proposed that the concentration of HDL was negatively correlated with atherosclerosis, and this assumption was confirmed in animal models [10]. Subsequently, the Framingham study confirmed that HDL levels were inversely associated with the risk of CAD [11]. In the process of RCT, cholesterol is initially transported from arterial macrophages to HDL, thus maintaining intracellular cholesterol homeostasis, which is critical for the viability and function of macrophages [12]. Once macrophages fail to efficiently expel the remaining excess cholesterol, they will degenerate to foam cells. This triggers the local aggregation of pro-inflammatory cells, thereby providing an environment conducive to the progression of atherosclerotic plaques [13]. In view of the above, it is hypothesized that a higher HDL level may be associated with a lower cardiovascular risk.

To verify this hypothesis, several trials based on statin therapy, cholesteryl ester transfer protein (CETP)-inhibitors and niacin have been conducted, but their results are unsatisfactory [14–17]. Furthermore, the National Institute of Health (NIH) terminated the AIM-HIGH study 18 months earlier than planned, since niacin (the most effective drug on raising HDL levels) could not provide additional cardiovascular benefits when used in combination with statins [18]. To sum up, these studies show that a mere increase in HDL levels has no cardiovascular benefits.

Thus, the research focus has shifted towards HDL functionality rather than quantity. Cholesterol efflux capacity (CEC) indicates the ability of HDL to facilitate the removal of cholesterol from the lipid-filled macrophages [19]. CEC depends primarily on the cholesterol content in macrophages, the expression of various macrophage-mediated transporters and the lipid and protein compositions of HDL as an extracellular receptor [20]. Moreover, the preventive effect of CEC on the progression of atherosclerotic lesion has been confirmed in animal models [9]. Recently, CEC has also been measured in clinical cohorts. Several studies have suggested that physiological CEC is negatively correlated with the development of CAD, independent of the HDL cholesterol levels [21, 22]. This study systematically investigated the relationships of CEC with CAD and cardiovascular mortality. In addition, dose-response analyses were also conducted for CEC and the risks of CAD and cardiovascular mortality.

## Methods

### Literature search strategy

This study protocol and report were based on the “Preferred Reporting Items for Systematic Reviews and Meta-Analyses” (PRISMA) statement [23] and were registered in the NPLASY-International Platform of Registered Systematic Review and Meta-analysis Protocols under the identifier of INPLASY202170006. Relevant studies were identified from four electronic databases (PubMed, Embase database, Cochrane Library, and Web of Science) from 1980 to February 1st, 2022, without any language restriction. Two groups of medical subject terms were applied, including “cardiovascular diseases” and “cholesterol efflux capacity “. Furthermore, to identify additional studies, previous systematic reviews or meta-analyses were reviewed as well. The detailed search strategy is presented in Supplementary Materials (Additional file 1).

### Parameters and outcomes of interest

The exposure of interest was HDL-CEC. All studies reporting CEC were included, regardless of the specific laboratory or calculation methods. According to the definitions of the included original studies, CAD was defined as coronary stenosis ≥50% confirmed by coronary angiography or multi-slice coronary computed tomography, or history of myocardial infarction or angina confirmed by hospital or autopsy records. Cardiovascular mortality was defined as death due to any cardiovascular cause or sudden cardiac death. In this study, our endpoints included CAD and cardiovascular mortality risk.

### Inclusion and exclusion criteria

With reference to the PICOS (Population, Intervention, Control, Outcome, and Study) criteria [24], the study inclusion criteria were as follows:

1. studies conducted on the general population with the age over 18 years;
2. studies, whose exposure of interest was HDL-CEC;
3. the CEC values of non-CAD population in case-control studies were regarded as the control group;
4. the CEC values in cohort studies were divided by medians or into quartiles, and patients with the lowest CEC quartile or those with CEC below the median were enrolled into the reference group according to the calculation method of original publications;
5. studies, that reported the risk of CAD or cardiovascular mortality;
6. studies, that were limited to case-control, cohort studies, or randomized controlled trials;
7. studies reporting relevant effect values such as odds ratios (ORs), relative risks (RRs), or hazard ratios (HRs) together with respective 95% confidence intervals (CIs).

The study exclusion criteria were:

1. studies focusing on cancer, autoimmune diseases, familial hyperlipidemia, or renal failure;
2. studies where the HDL-CEC values could not be obtained or converted;
3. studies not reporting the risk of CAD or cardiovascular mortality;
4. cross-sectional studies;
5. studies with unavailable ORs, RRs, or HRs;
6. conference abstracts, case reports, or case series.

### Data extraction and quality assessment

A uniform list was prepared to collect related baseline characteristics, including first author, year of publication, country, sample size, proportion of males, age of patients on inclusion, follow-up, subjects, endpoints, donor cell line, labeled cholesterol, and cholesterol acceptor. In addition, the maximum covariate-adjusted ORs, RRs, or HRs were extracted. The quality of the included studies was assessed using the Newcastle Ottawa Scale (NOS), with a total score of 9 stars [24]. Studies with a score of ≥6 stars were considered to have a low risk of bias, while those with a score of < 6 stars were deemed to show a high risk of bias [25].

### Statistical analysis

Firstly, we assessed the difference in CEC value between CAD and non-CAD populations. For this purpose, the sample numbers, mean CEC values, and SD of CEC in CAD and non-CAD groups were extracted from the selected studies. Considering the heterogeneities in the experimental measurement methods, the standardized mean difference (SMD) was utilized for assessment. For studies reporting median values and interquartile ranges, with a large sample size (> 100/group), the median values were treated as means, and SD was calculated by 75th minus 25th percentiles divided by 1.35 [26], otherwise SD could not be estimated.

Secondly, we assessed the difference in cardiovascular risk between the highest and the lowest CEC value groups. In cohort studies, CEC values were divided according to quartiles or medians, where the quartile 1 (Q1) or less-than median group was regarded as the lowest group, while the quartile 4 (Q4) or over median group as the highest group. Broadly speaking, the differences between the different effect values (e.g., ORs, RRs and HRs) were very minor if the incidence of the outcome is low (< 10%) [27]. The cumulative results were expressed as ORs for conservative assessment, while subgroup analyses were also performed to find any difference between the three measures of association (OR, RR or HR) [28]. On the other hand, statistical heterogeneity was assessed using the I^2^ statistic, where I^2^ values of 25, 50, and 75% indicated low, moderate, and high inconsistencies, respectively [29]. If there was a high heterogeneity between studies, subgroup analyses (number of studies > 10) were performed to explore the possible sources of heterogeneity between groups. Further, sensitivity analyses were carried out in parallel by progressively eliminating one study each time, so as to assess the potential sources of heterogeneity and the stability of our pooled results. Moreover, the DerSimonian and Laird (DL) method for random-effects model was employed to estimate the pooled standardized mean difference (SMD) and ORs, as the former evaluated the pooled results more conservatively compared to the fixed-effects model. Also, if sufficient studies (*n* ≥ 10) were included [18], Begg’s and Egger’s tests were utilized to assess the potential risk of publication bias. Later, trim and fill analyses were performed if there was evidence of publication bias.

Thirdly, this study focused on assessing the dose-response analyses between CEC and cardiovascular outcomes. To maximize the available studies, the robust error meta-regression approach proposed by Xu and Doi [30] was employed to establish the potential dose-response relationship between CEC and CAD and cardiovascular mortality. In this “one-stage” framework, each of the included studies was considered as a cohort across the entire population. In general, the lowest dose category was required as a reference for the included studies. In this analysis, the potential nonlinear trends in three knots were tested using the restricted cubic splines, and nonlinear *p*-values were calculated by testing whether the coefficient of the second spline was equal to zero. When the nonlinear p-value was < 0.05, the nonlinear model was adopted; otherwise, the linear model was adopted. Furthermore, when studying open intervals, the amplitudes were assumed to be the same as the adjacent categories. All statistical analyses were conducted using the statistical program Stata 12.0E.

## Results

### Search results and study characteristics

**Fig. 1:**
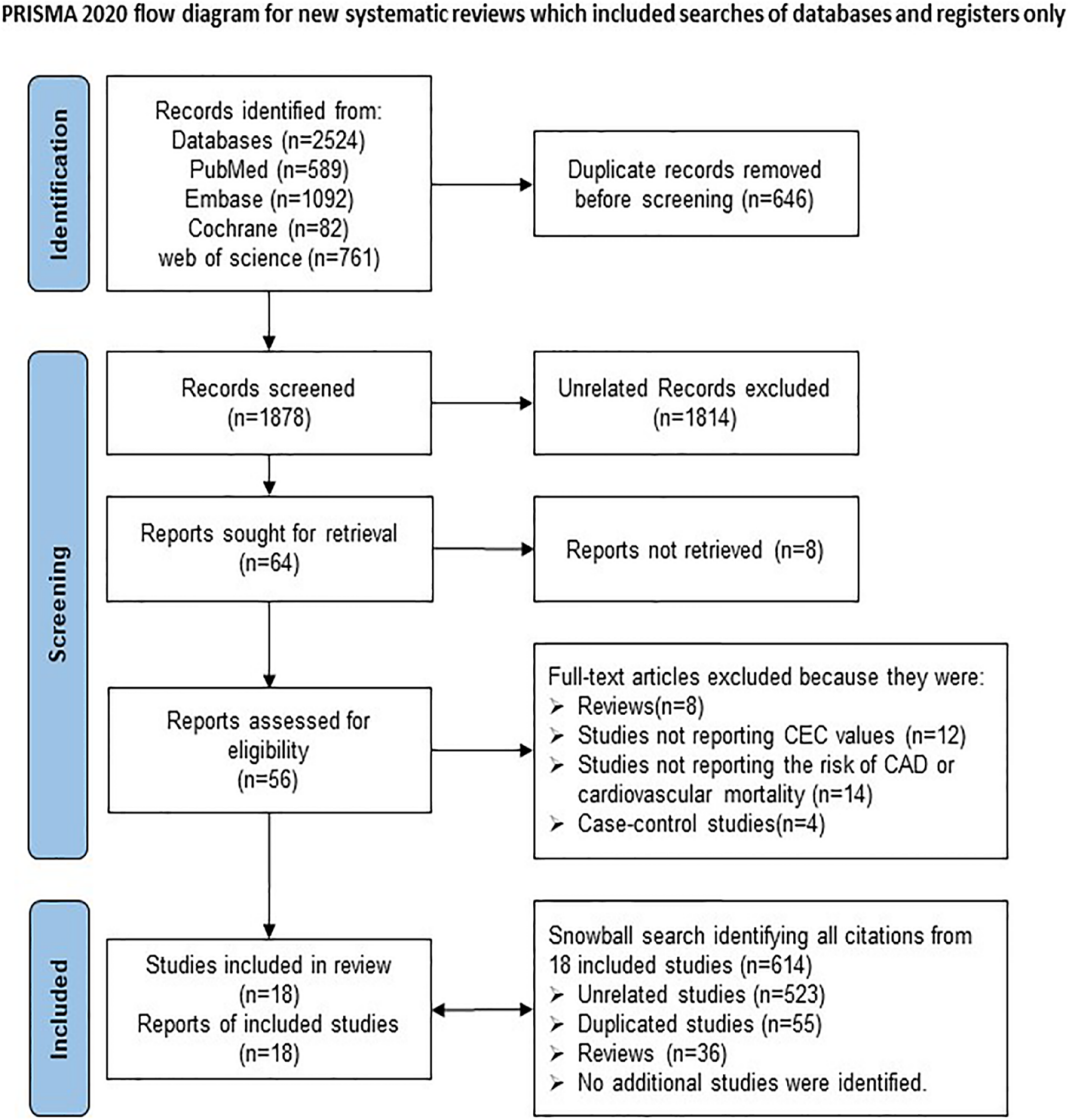
Flow chart of study selection for the meta-analysis

Initially, a total of 2524 studies were identified from the four databases. Among them, 646 records were identified more than once and consequently excluded, leaving 1878 studies. After screening the titles and abstracts of these 1878 studies, 1814 were excluded due to irrelevance, and 8 studies were excluded because of being not retrievable, leaving 56 studies for full-text review. Finally, 18 observational studies were included for the present meta-analysis. The specific reasons for study exclusion were as follows, a) reviews (*n* = 8); b) studies not reporting CEC (*n* = 12); c) studies not including the risk of CAD or cardiovascular mortality (*n* = 14); d) cross-sectional studies (*n* = 4). Further, a “snowball search” was performed based on the citation list of all included studies. In the 18 studies initially identified, there were 614 citations, whereas 523 were irrelevant for this meta-analysis, 55 were duplicates, and 36 were reviews. Ultimately, no additional studies were included in the analysis. The detailed flow chart of the study retrieval process is presented in Fig. 1.

Our meta-analysis included 14 case-control and four cohort studies [21, 22, 31–46] involving altogether 6298 cases. Eleven studies [21, 31–33, 37–40, 42, 43, 45], including 3521 cases, mentioned CEC differences between CAD and non-CAD groups. Additionally, nine studies [21, 22, 32–35, 40, 42, 46], involving 5023 cases, mentioned a relationship of CEC with CAD susceptibility, with seven studies [21, 22, 33, 34, 40, 42, 46] showing positive results and two studies [32, 35] showing no significant association. Four articles [22, 36, 41, 46], including 916 cases, reported the correlation between CEC and cardiovascular mortality risk, with two studies [36, 46] showing negative correlation and two studies [22, 41] showing no significant correlation. In the dose-response analyses, three studies [21, 22, 42] mentioned the dose-relationship between CEC and CAD risk, while another three [22, 36, 41] indicated the dose-relationship between CEC and cardiovascular mortality susceptibility. The detailed baseline information is presented in Table 1. The results of all the included studies were adjusted by HDL-C levels. Studies with a score of ≥6 stars were considered as high-quality studies (Supplementary Table 1).

**Table 1:** Characteristics of 18 observational studies

Author, year	Country	Sample Number	Proportion of males (%)	Age (years)	Follow-up (years)	Subjects	Endpoints	Donor Cell line	Labeling of cholesterol	Cholesterol acceptor
***Cohort studies***										
Rohatgi A [22], 2014	USA	132 cases	NA	30–65	Median 9.4	Non-CAD	1,2	J 774	BODIPY-C	ABDP
Liu C [36], 2016	China	122 cases	NA	40–85	3.8	CAD	2	J 774	BODIPY-C	ABDS
Ritsch A [41], 2020	Austria	448 cases	NA	62.8 ± 10.4	Median 9.9	Non-CAD	2	J 774	^3^ H-C	ABDS
Kuusisto S [34], 2019	UK	574 cases	NA	25–74	15	Non-CAD	1	J 774	^3^ H-C	ABDS
***Case-control studies***										
Saleheen D [42], 2015	USA	Case: 1745 Control: 1749	68	40–79	14	Case: CAD Control: non-CAD	1	J 774	^3^ H-C	ABDS
Zhang J [46], 2016	China	Case: 214 Control:116	78	67 ± 11	1	Case: CAD Control: non-CAD	1,2	J 774	^3^ H-C	ABDS
Li XM [35], 2013	China	Cohort A: Case 871 Control: 279 Cohort B: Case: 146 Control: 431	67 70	64 ± 11	3	Case: CAD Control: non-CAD	1	J 774	^3^ H-C	ABDS
Shea S [44], 2019	USA	Case: 270 Control: 270	NA	45–85	10	Case: CAD Control: non-CAD	1	THP-1 monocytes	^3^ H-C	UCH
Patel P [40], 2013	USA	Case: 23 Control: 46	74	58.2 ± 10	NA	Case: CAD Control: non-CAD	1	J 774	^3^ H-C	ABDS
Cahill L [32], 2019	USA	Case: 696 Control: 701	100	40–75	17	Case: CAD Control: non-CAD	1	J 774	^3^ H-C	ABDP
Ishikawa T [33], 2015	Japan	Case: 182 Control: 72	81.9	66.2 ± 10.3	2	Case: CAD Control: non-CAD	1	J 774	^3^ H-C	ABDS
Luo M [38], 2018	China	Case: 120 Control: 90	63.6	63.96 ± 7.85	NA	Case: CAD Control: non-CAD	1	THP-1 monocytes	^3^ H-C	ABDP
Khera A [21], 2011	USA	Case: 442 Control: 351	68.6	57 ± 9	NA	Case: CAD Control: non-CAD	1	J 774	^3^ H-C	ABDS
Wang G [45], 2018	China	Case: 40 Control: 40	62.5	30–75	2	Case: CAD Control: non-CAD	1	J 774	^3^ H-C	ABDS
Shao B [43], 2014	China	Case: 20 Control: 20	70	64 ± 11	10 months	Case: CAD Control: non-CAD	1	BHK	^3^ H-C	UCH
Luo M [37], 2017	China	Case: 140 Control: 99	66.4	63.10 ± 8.42	NA	Case: CAD Control: non-CAD	1	THP-1 monocytes	^3^ H-C	ABDP
Agarwala AP [31], 2015	USA	Case: 55 Control: 120	60	64 ± 11	NA	Case: CAD Control: non-CAD	1	J774	^3^ H-C	ABDP ABDS
Norimatsu K [39], 2017	Japan	Case: 58 Control: 146	69	61–73	NA	Case: CAD Control: non-CAD	1	J 774	^3^ H-C	UCH

*NA* not applicable. *CAD* Coronary artery diseases, *HDL-C* high-density lipoprotein cholesterol, *BODIPY-C* boron dipyrromethene difluoride-cholesterol, *ABDP* apolipoprotein B–depleted plasma, *ABDS* apolipoprotein B–depleted serum, *CAD* coronary artery disease, ^*3*^ *H-C* ^3^ H-cholesterol, *BHK* genetically modified baby hamster kidney cells, *UCH* HDL isolation by ultracentrifugation. 1, CAD incidence. 2, Cardiovascular mortality

### CEC values in CAD compared with non-CAD groups

**Fig. 2:**
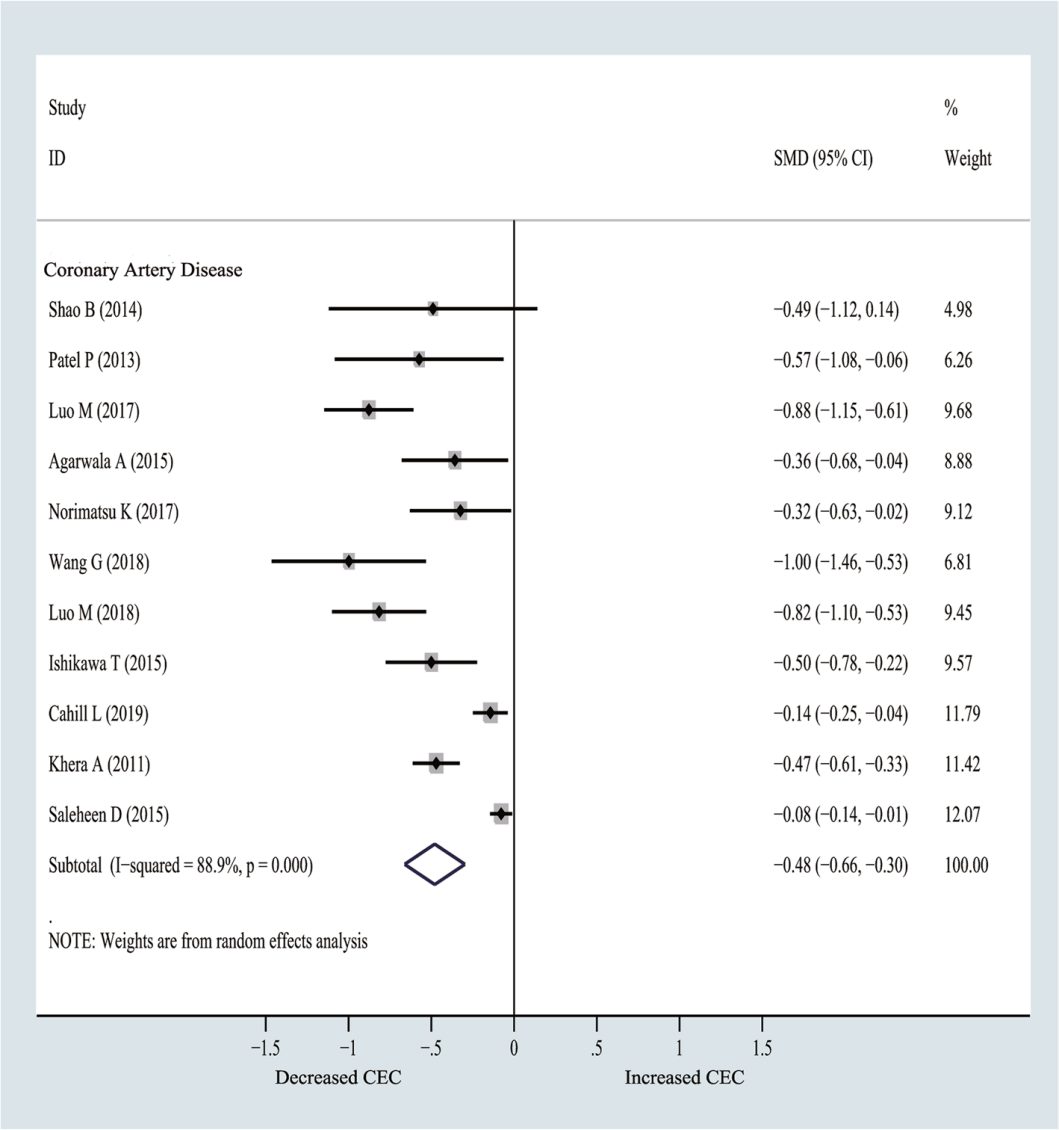
Forest plots of random-effects meta-analysis (between-study variance estimator: DerSimonian and Laird (DL)) for cholesterol efflux capacity (CEC) differences between coronary artery disease (CAD) vs. non-CAD group. Shown is the standardized mean difference (SMD) together with its 95% confidence interval (95% CI) as effect measure

CEC values of CAD group were significantly lower than those of non-CAD group (SMD = − 0.48, 95% CI − 0.66 to − 0.30; I^2^ = 88.9%) (Fig. 2). Sensitivity analyses were conducted by excluding one study each time, and the pooled results of CAD were slightly changed (Supplementary Fig. 1). Moreover, a funnel plot, which was prepared to assess the potential publication bias for CAD, exhibited asymmetry pointing towards a bias (Supplementary Fig. 2). Besides, there was no obvious evidence of publication bias (*p* = 0.350) detected by Begg’s test, whereas Egger’s tests suggested that there might be a publication bias (*p* = 0.007). Trim and fill analyses were thereby conducted because there were no additional studies. The pooled results obtained from the random model were the same.

### CEC and the risk of CAD (highest vs. lowest)

**Fig. 3:**
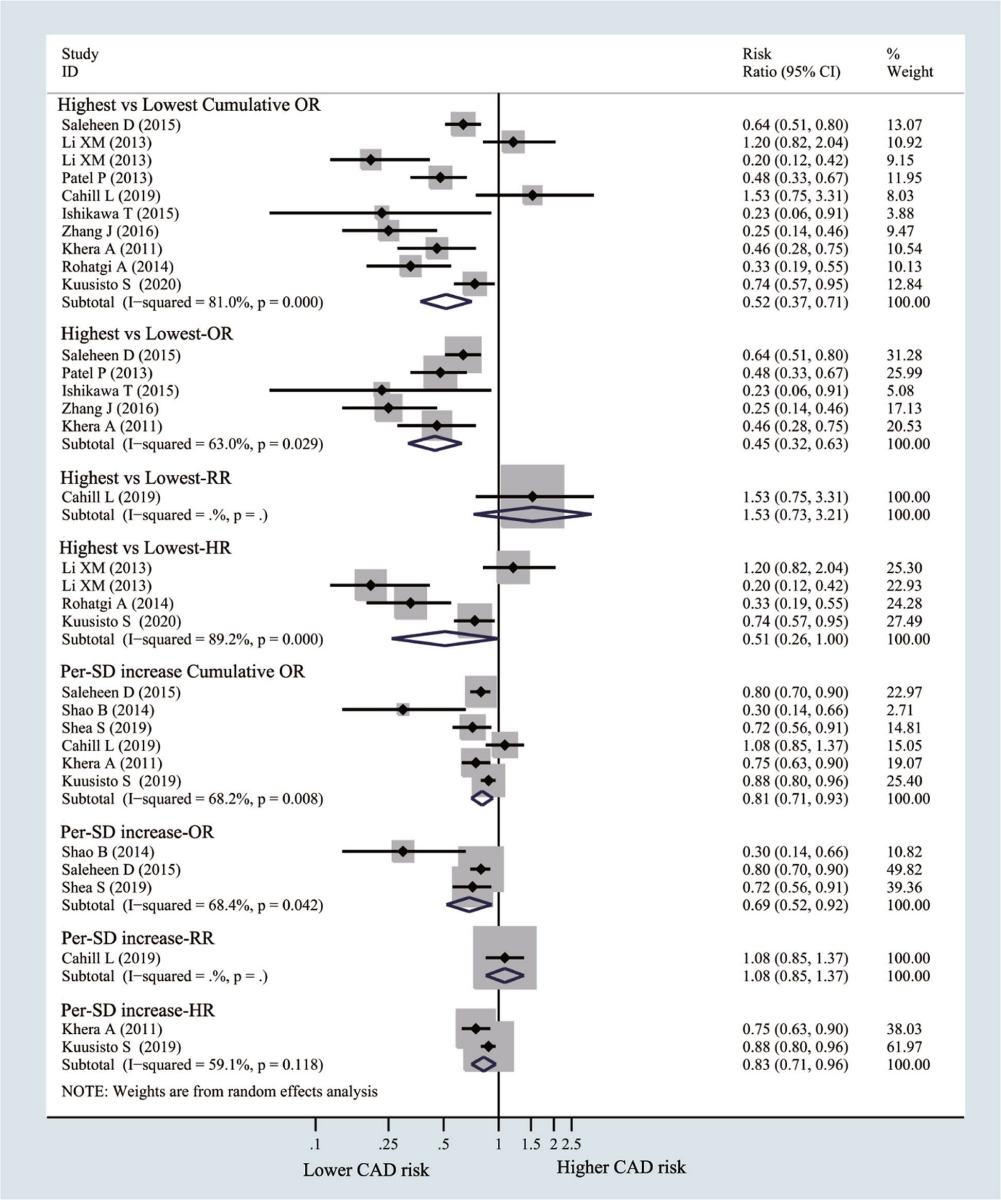
Forest plots of random-effects meta-analysis (between-study variance estimator: DerSimonian and Laird (DL)) for the differences of coronary artery disease (CAD) risk between highest cholesterol efflux capacity (CEC) vs. lowest CEC groups. Shown is the odds ratio (OR)/ relative risks (RR)/hazard ratio (HR) together with their 95% confidence interval (95% CI) as effect measure

The risk of CAD decreased by 48% in the highest CEC group compared with the lowest CEC group (OR = 0.52, 95% CI 0.37 to 0.71; I^2^ = 81%), and similar results were obtained after subgroup analysis by OR (OR = 0.45, 95% CI 0.32 to 0.63; I^2^ = 63%) and HR (HR = 0.51, 95% CI 0.26 to 1.0; I^2^ = 89.2%). Similarly, the risk of CAD decreased by 19% per 1-SD CEC increment (OR = 0.81, 95% CI 0.71 to 0.93; I^2^ = 68.2%), and similar results were observed from the subgroup analysis according to OR (OR = 0.69, 95% CI 0.52 to 0.92; I^2^ = 68.4%) and HR (HR = 0.83, 95% CI 0.71 to 0.96; I^2^ = 59.1%) (Fig. 3). In the sensitivity analysis, the pooled results remained stable after excluding one study each time (Supplementary Fig. 3A and B). No obvious evidence of publication bias was detected by Funnel plot (Supplementary Fig. 4), Begg’s test (*p* = 0.371) or Egger’s test (*p* = 0.283).

**Fig. 4:**
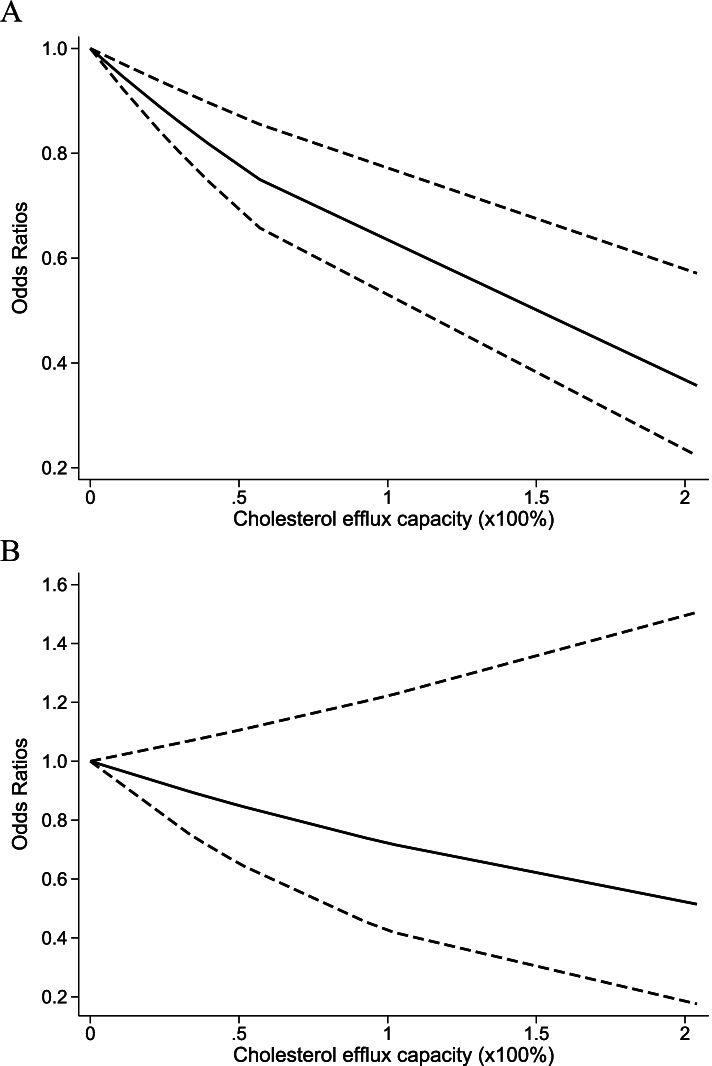
Dose-response analyses for cholesterol efflux capacity (CEC) and coronary artery disease (CAD) (**A**), and cardiovascular mortality (**B**). Shown is the odds ratio (OR) together with its 95% confidence interval (95% CI) as effect measure

As shown in Fig. 4A, a linear model was adopted to explore the association between CEC and the risk of CAD. As a result, the risk of CAD gradually decreased with the increase in CEC. Meanwhile, as CEC increased by 20%, the risk of CAD decreased by 10% (OR = 0.90; 95% CI 0.82–0.99).

### CEC and the risk of cardiovascular mortality (highest vs lowest)

**Fig. 5:**
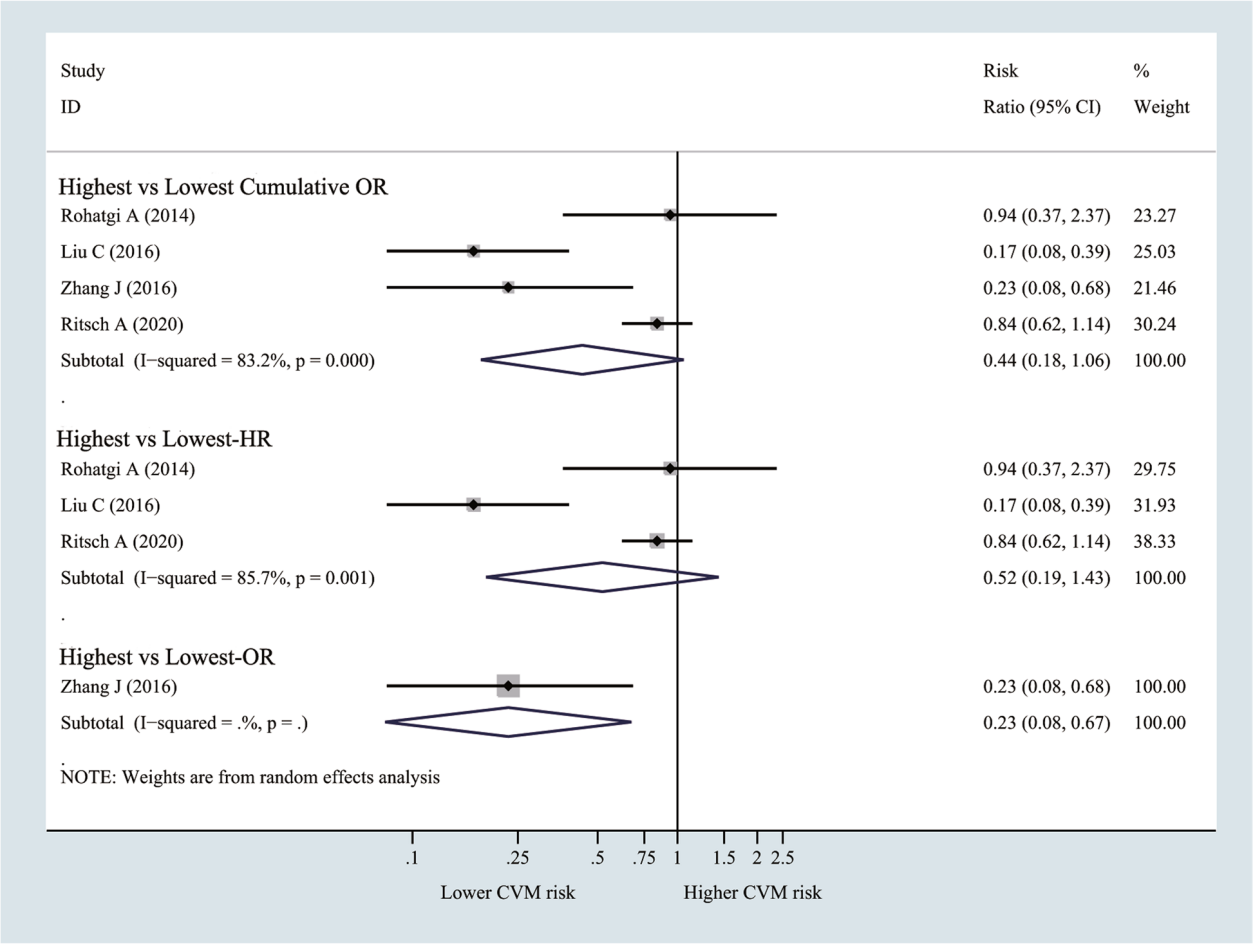
Forest plots of random-effects meta-analysis (between-study variance estimator: DerSimonian and Laird (DL)) for the differences of cardiovascular mortality risk between highest cholesterol efflux capacity (CEC) vs. lowest CEC groups. Shown is the odds ratio (OR)/ hazard ratio (HR) together with their 95% confidence interval (95% CI) as effect measure

It was observed from Fig. 5 that, the risk of cardiovascular mortality did not decrease in the highest CEC group compared with the lowest CEC group (OR = 0.44, 95% CI 0.18 to 1.06; I^2^ = 83.2%), similar results were obtained after subgroup analysis by HR (HR = 0.52, 95% CI 0.19 to 1.43; I^2^ = 85.7%). However, when the study by Ritsch et al. (the incidence of outcome was 18%>10%) was removed, the pooled results showed a weak correlation (OR = 0.33, 95% CI 0.11 to 0.96; *p* = 0.042) (Supplementary Fig. 5).

In Fig. 4B, the linear model was utilized to assess the correlation between CEC and the risk of cardiovascular mortality. As a result, the risk of cardiovascular mortality did not decrease with the increase in CEC.

### Subgroup analyses

To investigate the potential sources of heterogeneity in our study, subgroup analyses were conducted. However, due to the small number of studies included, we only performed subgroup analyses on the pooled results covering more than 10 studies, which included the following clinical characteristics: study design, country, year of publication, sample size, and study quality scores (Supplementary Table 1). Thereafter, the heterogeneity between studies was converted to zero based on subgroup analyses of the donor cell line and cholesterol acceptor suggesting that these factors might potentially contribute to the heterogeneity in the comparison of CEC between CAD and non-CAD groups. However, potential sources of heterogeneity between CEC and CAD risk were not identified.

## Discussion

The results of the meta-analysis can be summarized as follows: 1). CEC values were significantly lower in the CAD group compared to the non-CAD group. 2). The reduced CEC values were significantly associated with the risk of CAD. 3). There was no significant correlation between CEC values and the risk of cardiovascular mortality.

To the best of our knowledge, four previous systemic reviews and meta-analyses have systematically investigated the relationship of HDL-CEC with cardiovascular disease (CVD) [47–50]. Our meta-analysis includes seven [21, 22, 33, 35, 36, 42, 46], four [27, 33, 35],14 studies [21, 22, 31, 33, 36–40, 42–46] and 8 studies [21, 22, 32, 33, 35, 36, 42, 46] that were also included in these four meta-analyses, respectively. For instance, the study by Qiu et al. examined the associations of HDL-CEC with the risk of CVD and all-cause mortality. The results indicated that CEC was negatively correlated with the risk of CVD and possibly independent of the HDL level. The study by Lee et al. investigated that CEC is associated with adverse cardiovascular outcomes but does not increase the risk of all-cause and cardiovascular mortality, which is similar to our results. However, both of them also included cross-sectional studies, which might diminish the quality [51], as patients with renal failure and familial hyperlipidemia were also included, leading to a high cohort heterogeneity. Therefore, in view of this limitation, we restricted our analysis to cohort studies, case-control studies or randomized controlled trials. Patients in these case-control and cohort studies did not have a specific disease (cancer, autoimmune diseases, familial hyperlipidemia, or renal failure), and ORs were adjusted for covariates. Soria-Florido et al. investigated the potential associations of HDL function, including CEC values, the antioxidant and anti-inflammatory activities with the risks of CVD and mortality. They reported that higher CEC and anti-inflammatory activity were associated with the lower risks of CVD and mortality. These findings were in line with those obtained by Qiu et al. In addition, Ye et al. suggested that decreased CEC was an independent risk factor for CAD, which predicted the all-cause and cardiovascular mortality among CAD patients. Further, we added the information that CEC in the CAD population was generally reduced compared with that in the non-CAD population. Moreover, the risk of CAD significantly decreased in the high-CEC populations independent of HDL-C levels. However, the study by Li et al. reported the opposite result. In their study, two cohorts were included, one was an angiographic cohort and the other was an outpatient cohort. After 3 years of follow-up, the risk of myocardial infarction/stroke/death was inversely associated with CEC levels in the outpatient cohort and positively associated with CEC levels in the stable angiographic cohort, and these correlations remained significant even after adjustment for traditional cardiovascular risk factors. They considered that the presence of such results was related to specific cohort populations, that is, the relationship between CEC and CAD might differ significantly in different populations. Hence, the relationship between CEC and CAD might vary from person to person and more studies are necessary to prove this assumption.

HDL may delay the formation of atherosclerotic lesions through various mechanisms [52]. It is well known, that genetic variants that alter HDL-C levels are not necessarily associated with CAD risk, and that interventions aiming to increase HDL levels do not necessarily reduce cardiovascular events in CAD patients [53]. Thus, simply quantifying HDL levels is not a sufficient method to assess HDL function [54]. Whereas, CEC is involved in the reverse cholesterol transport from macrophages in the arterial wall to the liver, an important step in the process of HDL retardation of arterial plaque formation [55]. We thus suggest that HDL-CEC is indicative of plasma HDL function and can be taken as a biomarker to predict cardiovascular risk [56].

Further, HDL-CEC can exert anti-inflammatory and anti-atherosclerotic effects by inhibiting inflammatory processes in macrophages and activation of the inflammasome [57]. In this study, we observed that the risk of CAD obviously increased with the decrease of CEC, which may be related to the impaired HDL-CEC function in CAD patients. CAD patients frequently suffer from metabolic diseases [58]. Importantly, animal and in vitro experiments have shown that inflammation and metabolic disorders can convert HDL to a dysfunctional form. This not only impairs HDL’s CEC from macrophages and thus anti-atherosclerotic properties [59–61], but also can convert HDL to act pro-inflammatory, further increasing the risk of CAD [60, 62–64]. Therefore, alterations in HDL-CEC may be both a cause and a consequence in the development and progression of CAD. As outlined for CAD, also the incidence of cardiovascular mortality involves a multitude of traditional cardiovascular risk factors and genetic factors. CEC is only one factor amongst others and actually, in our study, we found no association between CEC and cardiovascular mortality. However, it is worth mentioning that CEC is a long-term stable trait being modestly independently heritable and independent of the HDL particle number, particle size and apolipoprotein A-II levels [56], thus functioning as a possible target for therapeutic intervention in the future.

Although CEC is a key indicator for the anti-atherosclerotic function of HDL and a strong predictor for CAD outcomes, measurement of HDL-CEC is not a part of routine lipid tests in CVD patients. This is partly explained by the lack of standardized procedures and the complexity of CEC mechanisms, because cholesterol efflux involves plenty of enzymes, like ATP-binding cassette transporter A1 (ABCA1), ATP-binding cassette transporter G1 (ABCG1), scavenger receptor class-BI (SR-BI), and aqueous diffusion [65]. Meanwhile, the determination of CEC value involves in-vitro testing, while standardized measurement of HDL-3 and HDL-2 levels can help to overcome technical limitations in routine testing. Cholesterol efflux to the HDL-3 subpopulation (350 kDa) is more efficient than to HDL-2 particles (175 kDa). Further, HDL-3 particles possess greater anti-oxidative and anti-apoptotic activities than HDL-2 particles. Finally, the increased HDL-2-to-HDL-3 ratio is associated with a higher risk of CAD [66, 67]. Therefore, determining the HDL-3-to-HDL-2 ratio may provide a simple standardized routine test to identify CAD manifestation or to predict outcome.

Compared to previous studies, the present study has the following strengths. To our knowledge, this is the first meta-analysis that systematically analyzes the association of CEC with the risks of CAD and cardiovascular mortality by dose-response analyses.

Nonetheless, certain limitations should also be noted. 1). The search and screening of this paper were done by the first author twice in July 2021 and February 2022, respectively. No kappa statistics were prepared. 2). There were high heterogeneities in the pooled cohorts used to generate the results of this paper, representing a limitation of this study. Heterogeneity is a consequence of the variability in experimental procedures, methods, study quality, and the differences in the characteristics of the studied patients. To address this problem, the donor cell line, labeled cholesterol, and cholesterol acceptors were identified as the possible sources of heterogeneity through subgroup analyses. Future experiments should consider the potential impact of experimental materials on the study. 3). Due to the insufficient number of existing studies, some pooled results were not further analyzed by subgroup analyses to detect the potential sources of heterogeneity. 4). A high percentage of patients were from the study by Ritsch et al.*,* where the incidence of cardiovascular mortality was exceptionally high (18%). This might be explained by the comparably long follow-up period (approximately 10 years) and the advanced age of the population. Except for the study by Ritsch et al., the other included cohort studies had a low incidence of cardiovascular outcomes (< 10%). Besides, although all the effect values of ORs/RRs included in this analysis were adjusted for covariates by the original authors (Supplementary Table 3), the effect of residual covariates on the results of this paper was not excluded. 5). The number of studies included in dose-response analyses was relatively small, and the results of this study should be interpreted with caution. Notably, results of dose-response analyses represent the trends in risk rather than precise quantification. 6). Most of the studies included in this paper are based on the assessment of serum efflux capacity, which may differ from the in vitro situation. Meanwhile, the CEC assay was performed in a closed system and ignored the dynamic comprehensive aspects of cholesterol flux through the RCT pathway. 7). Most of the cohorts included US and Asian patients, with only few Europeans and none from other regions. Considering the different genetic backgrounds, more multi-regional studies are warranted in the future. 8). The NOS score was used to evaluate the quality of cohort studies and case-control studies. Although the NOS score is currently widely accepted in meta-analysis, it is also criticized to deliver arbitrary results [68].

## Conclusions

Based on the current evidence, we suggest that a decreased CEC is strongly associated with the risk of CAD, independent of HDL-C level. However, a decreased CEC seems not to be related to cardiovascular mortality. In addition, CEC is negatively correlated with the risk of CAD in a linear manner. Nonetheless, further multi-center, multi-regional, and large sample studies are warranted in the future to complement and further validate these findings.

## Supplementary Information

**Additional file 1: Supplementary Figure 1.** The sensitivity analyses of CEC difference in coronary artery disease (CAD) and non-CAD group. CEC, cholesterol efflux capacity. The figure depicts pooled results of random-effect meta-analyses considering the remaining studies after excluding named study. **Supplementary Figure 2.** The funnel of CEC difference in coronary artery disease (CAD) and non-CAD group. CEC, cholesterol efflux capacity. **Supplementary Figure 3.** A The sensitivity analyses of CEC and the risk of coronary artery disease (CAD). CEC, cholesterol efflux capacity. The figure indicates the pooled results of the remaining studies after excluding named study (Study of Li MX includes two cohort). B. The sensitivity analyses of CEC and CAD risk by per 1-SD increasement. CEC, cholesterol efflux capacity. SD, standard deviation. **Supplementary Figure 4.** The funnel of CEC and CAD risk. CEC, cholesterol efflux capacity. CAD, coronary artery disease. **Supplementary Figure 5.** The sensitivity analyses of CEC and the risk of cardiovascular mortality. CEC, cholesterol efflux capacity. **Supplementary Table 1.** Subgroup Analyses. **Supplementary Table 2.** Quality Assessment of the 18 observational Studies. **Supplementary Table 3.** Characteristics of 18 Observational Studies.

## Data Availability

All data generated or analyzed during the current study are included in this published article and its supplementary information files.

## Abbreviations

CEC: Cholesterol efflux capacity
CAD: Coronary artery disease
RCT: Reverse cholesterol transport
CETP: Cholesteryl ester transfer protein
NIH: National Institute of Health
PRISMA: Preferred Reporting Items for Systematic Reviews and Meta-Analyses
SD: Standard deviation
OR: Odds ratios
RRs: Relative risks
HRs: Hazard ratios
Cis: Confidence intervals
SMD: Standardized mean difference
HDL-C: High-density lipoprotein cholesterol
CVD: Cardiovascular disease
NOS: Newcastle Ottawa Scale
ABCA1: ATP-binding cassette transporter A1
ABCG1: ATP-binding cassette transporter G1
DL: DerSimonian and Laird
Q1: Quartile 1
