# Supplementary material for: High-density lipoprotein cholesterol efflux capacity and incidence of coronary artery disease and cardiovascular mortality: a systematic review and meta-analysis
Source: Lipids Health Dis. 2022 May 28;21:47. doi: 10.1186/s12944-022-01657-3 (PMC9148501; doi:10.1186/s12944-022-01657-3)
Supplement: Supplementary file 1 — Additional file 1: Supplementary Figure 1. The sensitivity analyses of CEC difference in coronary artery disease (CAD) and non-CAD group. CEC, cholesterol efflux capacity. The figure depicts pooled results of random-effect meta-analyses considering the remaining studies after excluding named study. Supplementary Figure 2. The funnel of CEC difference in coronary artery disease (CAD) and non-CAD group. CEC, cholesterol efflux capacity. Supplementary Figure 3. A The sensitivity analyses of CEC and the risk of coronary artery disease (CAD). CEC, cholesterol efflux capacity. The figure indicates the pooled results of the remaining studies after excluding named study (Study of Li MX includes two cohort). B. The sensitivity analyses of CEC and CAD risk by per 1-SD increasement. CEC, cholesterol efflux capacity. SD, standard deviation. Supplementary Figure 4. The funnel of CEC and CAD risk. CEC, cholesterol efflux capacity. CAD, coronary artery disease. Supplementary Figure 5. The sensitivity analyses of CEC and the risk of cardiovascular mortality. CEC, cholesterol efflux capacity. Supplementary Table 1. Subgroup Analyses. Supplementary Table 2. Quality Assessment of the 18 observational Studies. Supplementary Table 3. Characteristics of 18 Observational Studies. [file 12944_2022_1657_MOESM1_ESM.docx]

**Supplementary materials**

***Supplementary Figure 1.*** The sensitivity analyses of CEC difference in coronary artery disease (CAD) and non-CAD group. CEC, cholesterol efflux capacity. The figure depicts pooled results of random-effect meta-analyses considering the remaining studies after excluding named study.


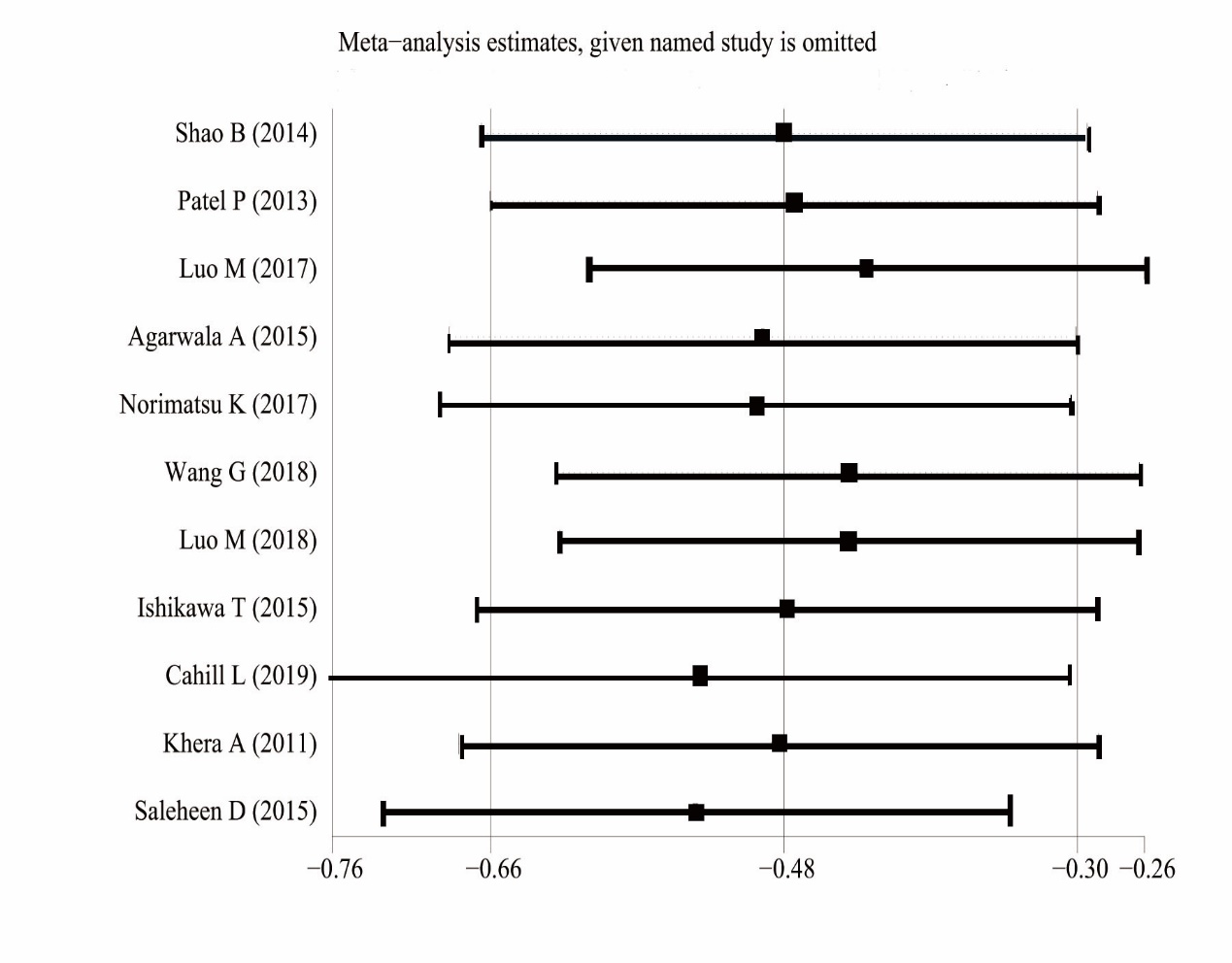


***Supplementary Figure 2.*** The funnel of CEC difference in coronary artery disease (CAD) and non-CAD group. CEC, cholesterol efflux capacity.


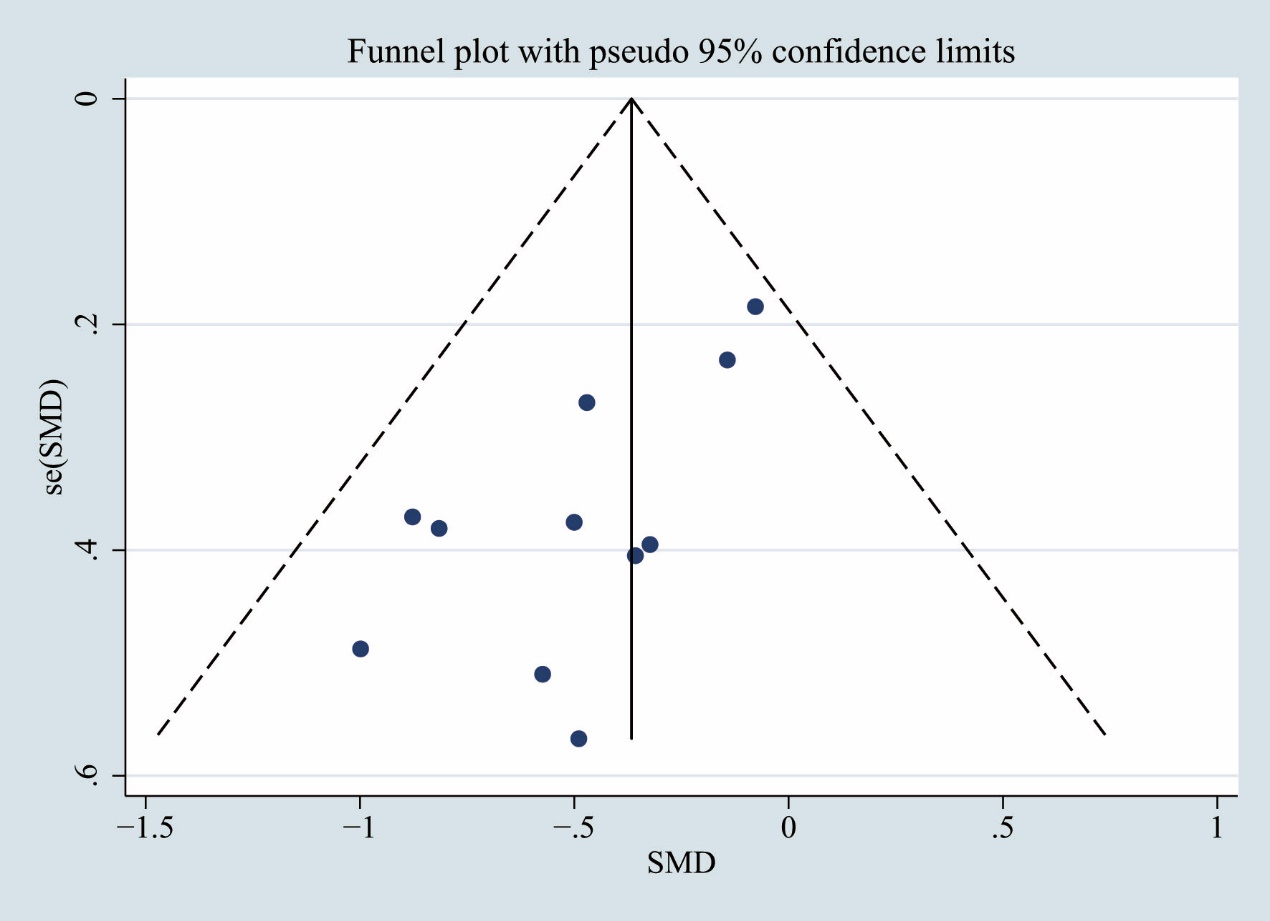


***Supplementary Figure 3A.*** The sensitivity analyses of CEC and the risk of coronary artery disease (CAD). CEC, cholesterol efflux capacity. The figure indicates the pooled results of the remaining studies after excluding named study (Study of Li MX includes two cohort).


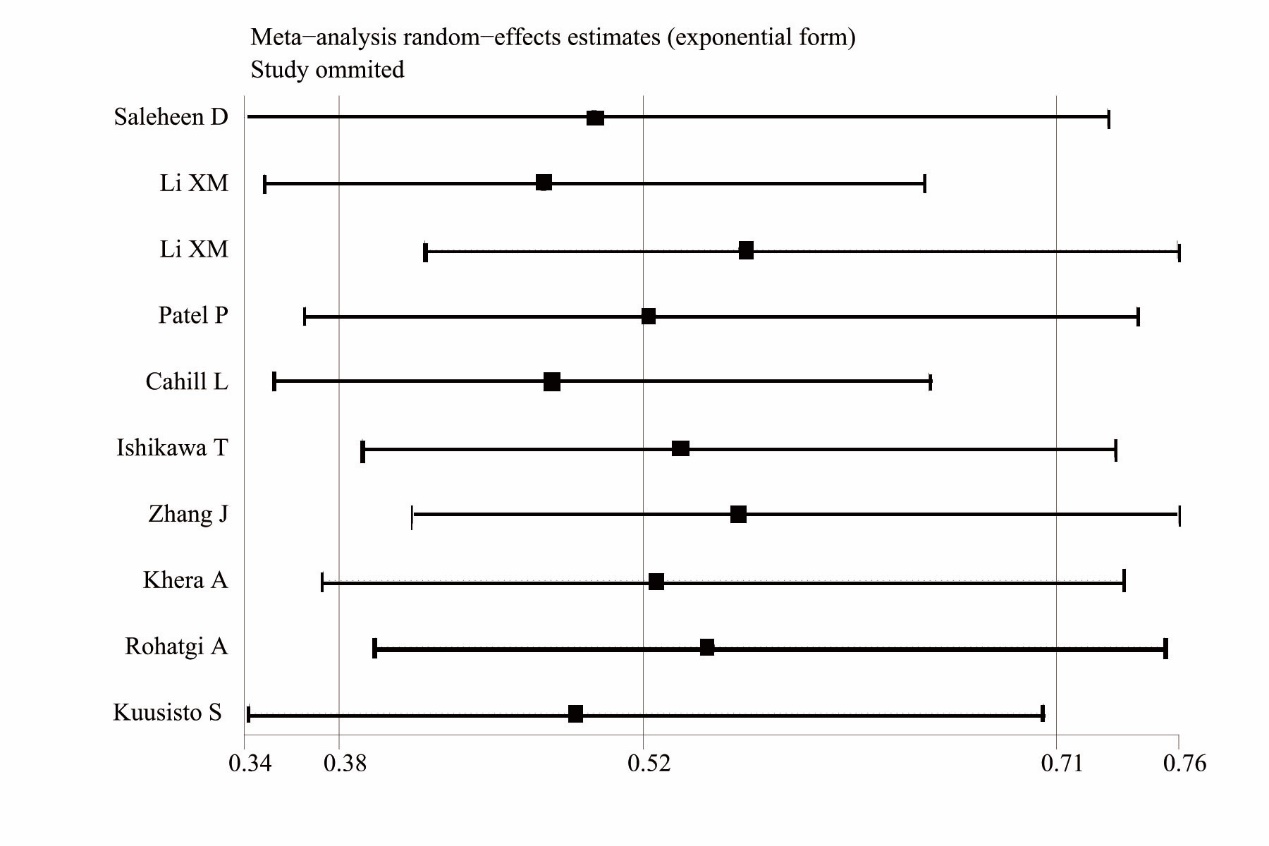


***Supplementary Figure 3B.*** The sensitivity analyses of CEC and CAD risk by per 1-SD increasement. CEC, cholesterol efflux capacity. SD, standard deviation.


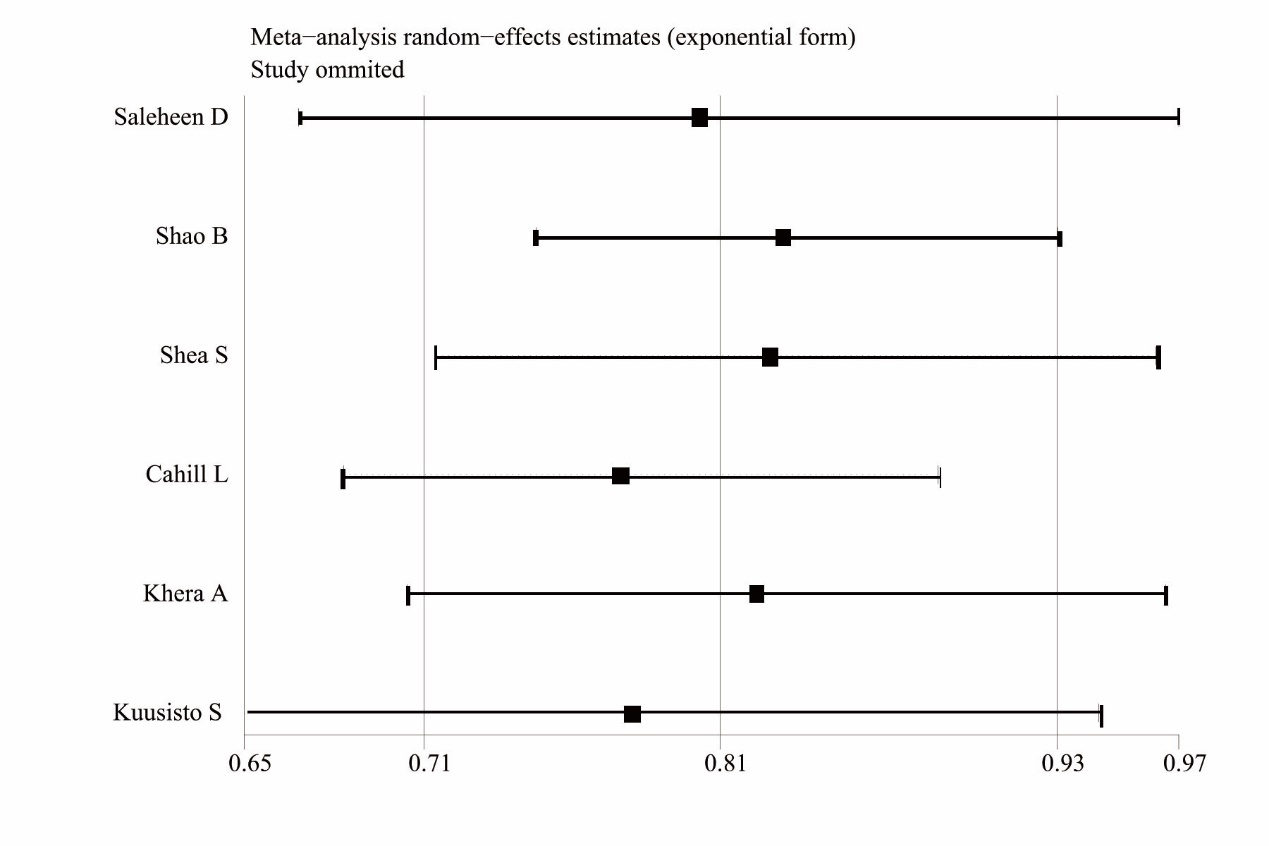


***Supplementary Figure 4.*** The funnel of CEC and CAD risk. CEC, cholesterol efflux capacity.

CAD, coronary artery disease.


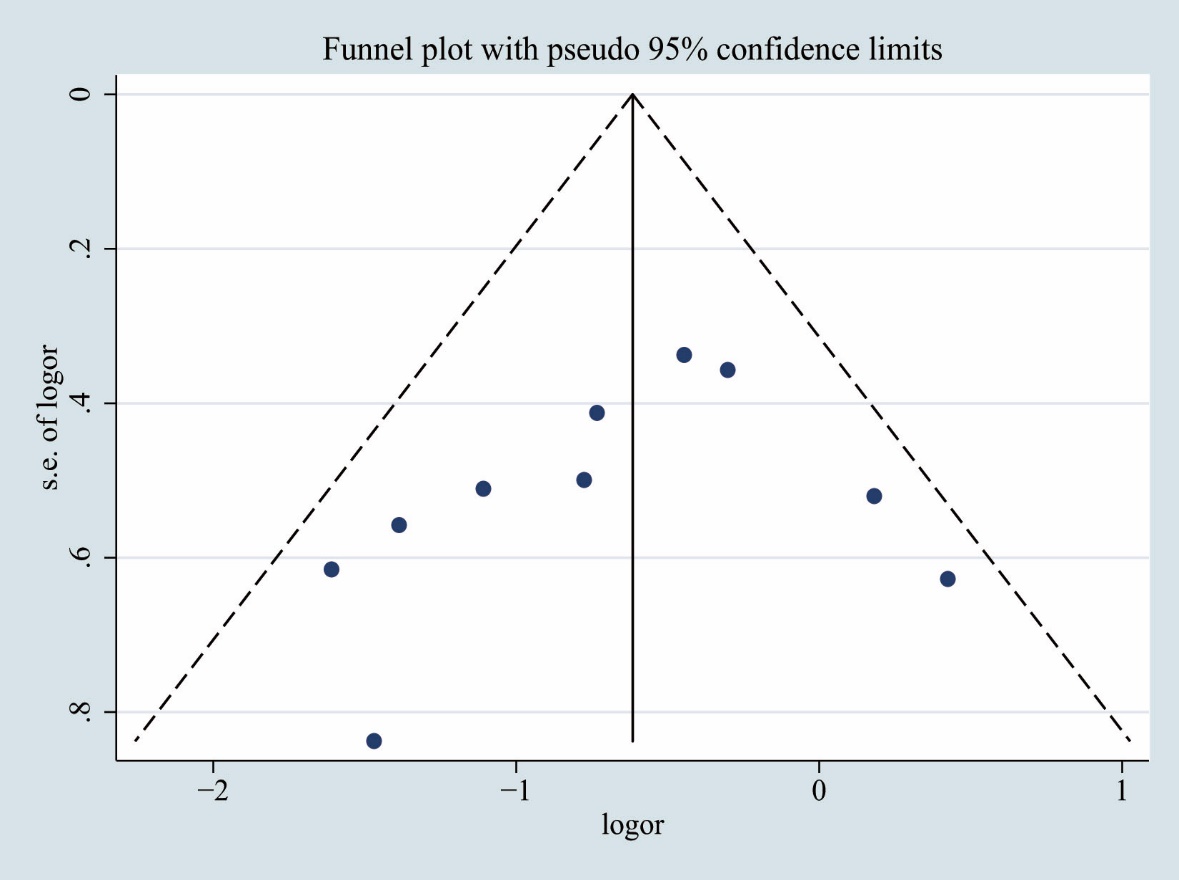


***Supplementary Figure 5*** The sensitivity analyses of CEC and the risk of cardiovascular mortality. CEC, cholesterol efflux capacity.


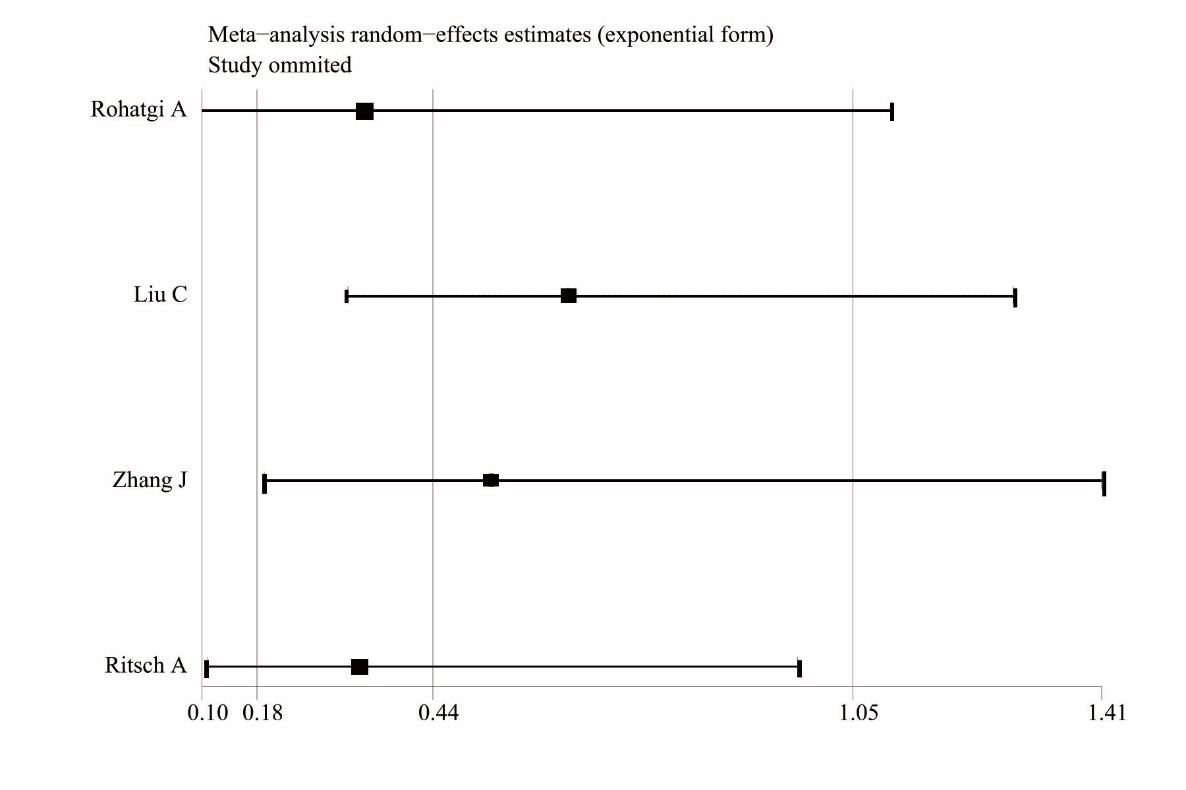


| **Supplementary Table 1. Subgroup Analyses** | | | | | | |
| --- | --- | --- | --- | --- | --- | --- |
|  | **CEC values**  **(CAD group vs non-CAD group)** | | | **CEC values and CAD Risk**  **（Highest vs Lowest）** | | |
| **Study Design** | n | SMD (95% CI) | I^2^ | n | OR (95% CI) | I^2^ |
| Cohort | 1 | - | - | 3 | 0.50(0.30-0.83) | 76.7% |
| Case-control | 10 | -0.48(-0.68, -0.28) | 88.2% | 7* | 0.52(0.32-0.83) | 84.5% |
| **Country** |  |  |  |  |  |  |
| America | 6 | -0.31(-0.48, -0.14) | 85.3% | 6 | 0.53(0.38-0.75) | 66.9% |
| Europe | 0 | - | - | 1 | - | - |
| Asia | 5 | -0.71(-0.96, -0.46) | 60.2% | 3* | 0.4(0.12-1.31) | 92.8% |
| **Published Year** |  |  |  |  |  |  |
| ≤2015 | 7 | -0.45(-0.70, -0.21) | 87.1% | 7* | 0.47(0.32-0.69) | 79.4% |
| ＞2015 | 4 | -0.53(-0.94, -0.12) | 92.3% | 3 | 0.65(0.28-1.50) | 87.4% |
| **Sample Size** |  |  |  |  |  |  |
| ≤300 | 8 | -0.51(-0.76, -0.27) | 81.8% | 4 | 0.48(0.23-1.01) | 80.1% |
| ＞300 | 3 | -0.43(-0.82, -0.05) | 95.5% | 6* | 0.54(0.37-0.78) | 83.0% |
| **Study quality** |  |  |  |  |  |  |
| 8-9 | 5 | -0.36(-0.58, -0.14) | 92.4% | 5 | 0.62(0.45-0.86) | 71.9% |
| 6-7 | 6 | -0.61(-0.81, -0.40) | 43.0% | 5* | 0.39(0.2-0.78) | 86.1% |
| **Donor Cell line** |  |  |  |  |  |  |
| J 774 | 8 | -0.37(-0.54, -0.2) | 85.1% | 10* | 0.52(0.37-0.71) | 81% |
| THP-1 | 2 | -0.85(-1.04, -0.65) | **0%** | 0 | - | - |
| Others | 1 | - |  |  |  |  |
| **Labeled-cholesterol** |  |  |  |  |  |  |
| ^3^ H-C | 11 | -0.48(-0.66, -0.30) | 88.9% | 9* | 0.54(0.39-0.76) | 81.2% |
| BODIPY-C | 0 | - | - | 1 | - |  |
| **Cholesterol acceptor** |  |  |  |  |  |  |
| ABDP | 3 | -0.6(-1.15, -0.05) | 94.9% | 8* | 0.49(0.35-0.69) | 80.7% |
| ABDS | 5 | -0.48(-0.77, -0.18) | 91% | 2 | 0.69(0.15-3.12) | 90.8% |
| Others | 3 | -0.36(-0.57, -0.15) | **0%** | 0 | - |  |

* The study of Li XM et al. included two cohorts, which were analyzed separately. CAD, coronary artery disease. SMD, standardized mean difference. OR, odds ratio.

| **Supplementary Table 2. Quality Assessment of the 18 observational Studies** | | | | |
| --- | --- | --- | --- | --- |
| **Study**  **(First Author, Year)** | **Selection** | **Comparability** | **Outcome/ exposure** | **Total** |
| Rohatgi A,2014 | **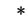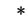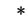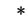** | **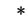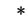** | **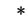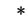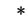** | 9 |
| Liu C, 2016 | **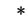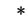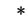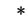** | **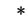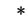** | **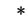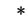** | 8 |
| Ritsch A, 2020 | **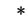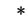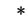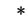** | **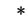** | **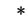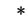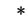** | 8 |
| Kuusisto S, 2019 | **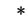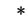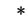** | **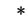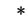** | **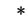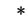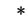** | 8 |
| Saleheen D, 2015 | **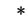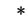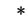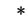** | **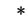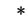** | **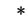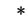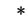** | 9 |
| Zhang J, 2016 | **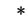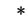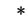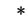** | **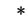** | **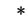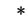** | **7** |
| Li XM, 2013 | **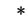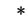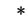** | **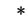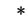** | **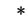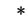** | **7** |
| Shea S, 2019 | **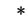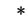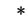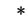** | **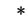** | **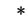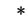** | **7** |
| Patel P, 2013 | **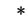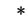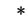** | **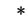** | **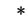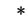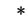** | **7** |
| Cahill L, 2019 | **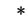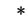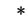** | **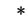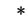** | **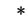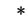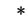** | 8 |
| Ishikawa T ,2015 | **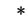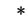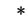** | **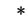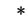** | **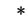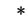** | **7** |
| Luo M, 2018 | **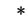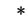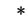** | **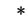** | **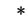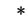** | 6 |
| Khera A, 2011 | **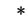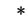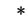** |  |  | 8 |
| Wang G, 2018 |  |  |  | 7 |
| Shao B, 2014 |  |  |  | 7 |
| Luo M, 2017 |  |  |  | 8 |
| Agarwala A, 2015 |  |  |  | 7 |
| Norimatsu K, 2017 |  |  |  | 6 |

| **Supplementary table 3. Characteristics of 18 Observational Studies** | | | |
| --- | --- | --- | --- |
| **Author, year** | **Endpoints** | **OR/RR/HR**  **(95% CI)** | **Maximum Adjusted Covariates** |
| Rohatgi A, 2014 | 1,2 | Quartile 4 vs. Quartile 1  0.26 (0.16-0.42) | age, sex, body mass index, alcohol intake smoking status diabetes mellitus, hypertension, use of lipid-lowering drugs, total cholesterol and triglycerides, density lipoprotein cholesterol, urinary albumin excretion, estimated glomerular filtration rate |
| Liu C, 2016 | 2 | Quartile 4 vs. Quartile 1  0.17(0.08-0.39) | age, gender, body mass index, smoking and alcohol drinking, prevalence of hypertension, diabetes mellitus, dyslipidemia, and use lipid-lowering drugs, total plasma cholesterol, triglycerides(log-transformed), LDL cholesterol, HDL cholesterol, and Apolipoprotein A-I levels |
| Ritsch A, 2020 | 2 | Quartile 4 vs. Quartile 1  0.84(0.62-1.14) | age, gender, use of statins, cardiovascular artery diseases, diabetes mellitus, smoking, triglycerides, LDL cholesterol, HDL cholesterol, adiponectin, fibrinogen, and C-reactive protein |
| Kuusisto S, 2019 | 1 | Quartile 4 vs. Quartile 1  0.74(0.57-0.95) | age, sex, geographical region, diabetes, mean arterial blood pressure, blood pressure treatment, smoking, log BMI, total cholesterol, log TG (triglycerides), lipid lowering treatment, HDL |
| Saleheen D, 2015 | 1 | Tertile 3 vs. Tertile 1  0.64 (0.51-0.80) | age, sex, batch number, diabetes, hypertension, alcohol use, hip ratio, BMI, LDL cholesterol, HDL |
| Zhang J, 2016 | 1,2 | CEC＞1.3 vs. CEC≤1.3  0.25(0.14-0.46) | age, sex, hypertension, diabetes, current smoking, serum LDL cholesterol, HDL cholesterol, Apo A, Apo B, and regular medication, Angiotensin I |
| Li XM, 2013 | 1 | stable angiographic cohort:  Tertile 3 vs. Tertile 1 1.2(0.82=2.04)  Outpatient cohort:  Tertile 3 vs. Tertile 1  0.2 (0.12-0.42) | age, sex, smoking, diabetes mellitus, hypertension, low-density lipoprotein-cholesterol, high-density lipoprotein-cholesterol levels |
| Shea S, 2019 | 1 | CAD: Per 1-SD increase  0.72 (0.56–0.91) | age, sex, race/ethnicity, body mass index, site, diabetes mellitus status, current smoking, total  and HDL cholesterol, statin use, hypertension medication, and systolic blood pressure, pack-years smoking, alcohol use, intentional exercise, Mediterranean diet |
| Patel P, 2013 | 1 | Highest vs. lowest  0.48(0.33-0.67) | age, blood pressure, creatinine, total  cholesterol, LDL, HDL mass |
| Cahill L, 2019 | 1 | Quintile 5 vs. Quintile 1  1.53(0.75-3.11) | age, smoking, and month of blood draw, BMI, fasting status, history of hypertension, history of hypercholesterolemia, parental CAD before age 60, and alcohol intake, LDL-C, log TG, HDL |
| Ishikawa T, 2015 | 1 | Highest vs. lowest  0.23(0.06-0.91) | age, sex, hypertension, dyslipidemia, diabetes, previous CVD, TC, HDL, Statin administration |
| Luo M, 2018 | 1 | CAD: 14.58 ± 2.06% (Mean ±SD)  Non-CAD: 12.51±2.83% (Mean ±SD) | NA |
| Khera A, 2011 | 1 | Quartile 4 vs. Quartile 1  0.46 (0.28–0.75) | age, sex, smoking status, presence or absence of diabetes, presence or absence of hypertension, low-density lipoprotein cholesterol, HDL, Apolipoprotein A-I |
| Wang G, 2018 | 1 | CAD: 11.13±1.53(Mean ±SD)  Non-CAD: 12.76±1.70(Mean ±SD) | NA |
| Shao B, 2014 | 1 | per 1-SD increase  0.3(0.14-0.66) | HDL |
| Luo M, 2017 | 1 | CAD: 12.5 ± 2.8% (Mean ±SD)  Non-CAD: 14.7 ± 2.0% (Mean ±SD) | NA |
| Agarwala A, 2015 | 1 | CAD: 1.96 ± 0.39(Mean ±SD)  Non-CAD: 2.11 ± 0.43(Mean ±SD) | NA |
| Norimatsu K, 2017 | 1 | CAD: 12.9 ±4 (Mean ±SD)  Non-CAD: 11.5 ± 5 (Mean ±SD) | NA |

1, CAD. 2, cardiovascular mortality. HDL, high-density lipoprotein. BMI, body mass index. CVD, cardiovascular diseases. NA, not applicable. TC, total cholesterol. LDL, low-density lipoprotein. TG, triglycerides.

**Appendix 1**

**PubMed**

1. Search: (((("cardiovascular diseases"[Mesh]) OR (cardiovascular disease)) OR (Disease, Cardiovascular)) OR (Diseases, Cardiovascular))
2. Search: Cholesterol Efflux Capacity
3. 1 and 2 589

**Web of Science**

1. (ALL= (cardiovascular diseases) OR ALL= (cardiovascular disease) OR ALL= (Disease, Cardiovascular) OR ALL= (Diseases, Cardiovascular))

2. (ALL=（Cholesterol Efflux Capacity）)

3. 1 and 2 761

**Embase**

1. 'Cardiovascular disease'/exp
2. angiocardiopathy OR (angiocardiovascular AND disease) OR (cardiovascular AND complication) OR (cardiovascular AND diseases) OR (cardiovascular AND disorder) OR (cardiovascular AND disturbance) OR (cardiovascular AND lesion) OR (cardiovascular AND syndrome) OR (cardiovascular AND vegetative AND disorder) OR (complication, AND cardiovascular) OR (disease, AND cardiovascular) OR (major AND adverse AND cardiovascular AND event)
3. 1 or 2
4. cholesterol AND efflux AND capacity
5. 3 and 4 1092

**Cochrane**

1. MeSH descriptor: [cardiovascular diseases] explode all trees
2. (Diseases, Cardiovascular): ti,ab,kw OR (Disease, Cardiovascular):ti,ab,kw OR (Cardiovascular Disease):ti,ab,kw
3. #1 or #2
4. (Cholesterol Efflux Capacity)
5. #3 and #4 82

**PRISMA checklist**

| **Section and Topic** | **Item #** | **Checklist item** | **Location where item is reported** |
| --- | --- | --- | --- |
| **TITLE** | | |  |
| Title | 1 | Identify the report as a systematic review. | Page 1 |
| **ABSTRACT** | | |  |
| Abstract | 2 | See the PRISMA 2020 for Abstracts checklist. | Page 2 |
| **INTRODUCTION** | | |  |
| Rationale | 3 | Describe the rationale for the review in the context of existing knowledge. | Page 3-4 |
| Objectives | 4 | Provide an explicit statement of the objective(s) or question(s) the review addresses. | Page 4 |
| **METHODS** | | |  |
| Eligibility criteria | 5 | Specify the inclusion and exclusion criteria for the review and how studies were grouped for the syntheses. | Page 4-5 |
| Information sources | 6 | Specify all databases, registers, websites, organisations, reference lists and other sources searched or consulted to identify studies. Specify the date when each source was last searched or consulted. | Page 4 |
| Search strategy | 7 | Present the full search strategies for all databases, registers and websites, including any filters and limits used. | Page 4 |
| Selection process | 8 | Specify the methods used to decide whether a study met the inclusion criteria of the review, including how many reviewers screened each record and each report retrieved, whether they worked independently, and if applicable, details of automation tools used in the process. | Page 4 |
| Data collection process | 9 | Specify the methods used to collect data from reports, including how many reviewers collected data from each report, whether they worked independently, any processes for obtaining or confirming data from study investigators, and if applicable, details of automation tools used in the process. | Page 4 |
| Data items | 10a | List and define all outcomes for which data were sought. Specify whether all results that were compatible with each outcome domain in each study were sought (e.g. for all measures, time points, analyses), and if not, the methods used to decide which results to collect. | Page 5-6 |
|  | 10b | List and define all other variables for which data were sought (e.g. participant and intervention characteristics, funding sources). Describe any assumptions made about any missing or unclear information. | Page 5 |
| Study risk of bias assessment | 11 | Specify the methods used to assess risk of bias in the included studies, including details of the tool(s) used, how many reviewers assessed each study and whether they worked independently, and if applicable, details of automation tools used in the process. | Page 5 |
| Effect measures | 12 | Specify for each outcome the effect measure(s) (e.g. risk ratio, mean difference) used in the synthesis or presentation of results. | Page 6 |
| Synthesis methods | 13a | Describe the processes used to decide which studies were eligible for each synthesis (e.g. tabulating the study intervention characteristics and comparing against the planned groups for each synthesis (item #5)). | Page 4-5 |
|  | 13b | Describe any methods required to prepare the data for presentation or synthesis, such as handling of missing summary statistics, or data conversions. | Page 6 |
|  | 13c | Describe any methods used to tabulate or visually display results of individual studies and syntheses. | Page 6 |
|  | 13d | Describe any methods used to synthesize results and provide a rationale for the choice(s). If meta-analysis was performed, describe the model(s), method(s) to identify the presence and extent of statistical heterogeneity, and software package(s) used. | Page 6 |
|  | 13e | Describe any methods used to explore possible causes of heterogeneity among study results (e.g. subgroup analysis, meta-regression). | Page 6 |
|  | 13f | Describe any sensitivity analyses conducted to assess robustness of the synthesized results. | Page 6 |
| Reporting bias assessment | 14 | Describe any methods used to assess risk of bias due to missing results in a synthesis (arising from reporting biases). | Page 6 |
| Certainty assessment | 15 | Describe any methods used to assess certainty (or confidence) in the body of evidence for an outcome. | Page 6 |
| **RESULTS** | | |  |
| Study selection | 16a | Describe the results of the search and selection process, from the number of records identified in the search to the number of studies included in the review, ideally using a flow diagram. | Page 6 |
|  | 16b | Cite studies that might appear to meet the inclusion criteria, but which were excluded, and explain why they were excluded. | Page 6-7 |
| Study characteristics | 17 | Cite each included study and present its characteristics. | Page 7 |
| Risk of bias in studies | 18 | Present assessments of risk of bias for each included study. | Page 7 |
| Results of individual studies | 19 | For all outcomes, present, for each study: (a) summary statistics for each group (where appropriate) and (b) an effect estimate and its precision (e.g. confidence/credible interval), ideally using structured tables or plots. | Page 7-8 |
| Results of syntheses | 20a | For each synthesis, briefly summarise the characteristics and risk of bias among contributing studies. | Page 7-8 |
|  | 20b | Present results of all statistical syntheses conducted. If meta-analysis was done, present for each the summary estimate and its precision (e.g. confidence/credible interval) and measures of statistical heterogeneity. If comparing groups, describe the direction of the effect. | Page 7-8 |
|  | 20c | Present results of all investigations of possible causes of heterogeneity among study results. | Page 8 |
|  | 20d | Present results of all sensitivity analyses conducted to assess the robustness of the synthesized results. | Page 7-8 |
| Reporting biases | 21 | Present assessments of risk of bias due to missing results (arising from reporting biases) for each synthesis assessed. | Page 7-8 |
| Certainty of evidence | 22 | Present assessments of certainty (or confidence) in the body of evidence for each outcome assessed. | Page 7-8 |
| **DISCUSSION** | | |  |
| Discussion | 23a | Provide a general interpretation of the results in the context of other evidence. | Page 8-10 |
|  | 23b | Discuss any limitations of the evidence included in the review. | Page 11 |
|  | 23c | Discuss any limitations of the review processes used. | Page 11 |
|  | 23d | Discuss implications of the results for practice, policy, and future research. | Page 10 |
| **OTHER INFORMATION** | | |  |
| Registration and protocol | 24a | Provide registration information for the review, including register name and registration number, or state that the review was not registered. | Page 4 |
|  | 24b | Indicate where the review protocol can be accessed, or state that a protocol was not prepared. | Page 4 |
|  | 24c | Describe and explain any amendments to information provided at registration or in the protocol. | Page 4 |
| Support | 25 | Describe sources of financial or non-financial support for the review, and the role of the funders or sponsors in the review. | Page 12 |
| Competing interests | 26 | Declare any competing interests of review authors. | Page 12 |
| Availability of data, code and other materials | 27 | Report which of the following are publicly available and where they can be found: template data collection forms; data extracted from included studies; data used for all analyses; analytic code; any other materials used in the review. | Page 12 |
